# STC3141 improves acute lung injury through neutralizing circulating histone in rat with experimentally-induced acute respiratory distress syndrome

**DOI:** 10.3389/fphar.2023.1166814

**Published:** 2023-05-22

**Authors:** Yangyang Ge, Chenchen Wang, Chenye Yao, Yong Wang, Yuduo Zheng, Junjie Luo, Jiayi Chen, Yu Wang, Fuquan Wang, Li Wang, Yun Lin, Lin Shi, Shanglong Yao

**Affiliations:** ^1^ Department of Anesthesiology, Union Hospital, Tongji Medical College, Huazhong University of Science and Technology, Wuhan, China; ^2^ Institute of Anesthesia and Critical Care Medicine, Union Hospital, Tongji Medical College, Huazhong University of Science and Technology, Wuhan, China; ^3^ Department of Neurology, Union Hospital, Tongji Medical College, Huazhong University of Science and Technology, Wuhan, China; ^4^ Grand Pharma (China) Co., Ltd, Hubei, China

**Keywords:** ARDS, ALI, histone, STC3141, histone neutralization

## Abstract

**Background:** Acute respiratory distress syndrome (ARDS) remains a challenge because of its high morbidity and mortality. Circulation histones levels in ARDS patients were correlated to disease severity and mortality. This study examined the impact of histone neutralization in a rat model of acute lung injury (ALI) induced by a lipopolysaccharide (LPS) double-hit.

**Methods:** Sixty-eight male Sprague-Dawley rats were randomized to sham (N = 8, received saline only) or LPS (N = 60). The LPS double-hit consisted of a 0.8 mg/kg intraperitoneal injection followed after 16 h by 5 mg/kg intra-tracheal nebulized LPS. The LPS group was then randomized into five groups: LPS only; LPS +5, 25, or 100 mg/kg intravenous STC3141 every 8 h (LPS + L, LPS + M, LPS + H, respectively); or LPS + intraperitoneal dexamethasone 2.5 mg/kg every 24 h for 56 h (LPS + D). The animals were observed for 72 h.

**Results:** LPS animals developed ALI as suggested by lower oxygenation, lung edema formation, and histological changes compared to the sham animals. Compared to the LPS group, LPS + H and +D groups had significantly lower circulating histone levels and lung wet-to-dry ratio, and the LPS + D group also had lower BALF histone concentrations; the blood neutrophils and platelets counts in LPS + D group did not change, meanwhile, the LPS + L, +M and +H groups had significantly lower neutrophil counts and higher platelet counts in the blood; the total number of BALF WBC, platelet counts, MPO and H3 were significantly lower in the LPS + L, +M, +H and +D groups than in the LPS only group; and the degree of inflammation was significantly less in the LPS + L, +M, +H and +D groups, moreover, inflammation in the LPS + L, +M and +H animals showed a dose-dependent response; finally, the LPS + L, +M, +H and +D groups had improved oxygenation compared to the LPS group, and there were no statistical differences in PCO2 or pH among groups. All animals survived.

**Conclusion:** Neutralization of histone using STC3141, especially at high dose, had similar therapeutic effects to dexamethasone in this LPS double-hit rat ALI model, with significantly decreased circulating histone concentration, improved acute lung injury and oxygenation.

## 1 Introduction

Acute respiratory distress syndrome (ARDS), characterized by bilateral chest radiographically opacities and severe hypoxemia (Force and V Marco Ranieri GDRB, 2012), remains a major challenge with its high morbidity and mortality. In an observational study across 50 countries and involving 459 intensive care units (ICU), ARDS was responsible for 10% of ICU admissions and 23% of mechanically ventilated patients; mortality was 35% and could be as high as 46% in severe ARDS (Bellani et al., 2016). Currently, only therapies that have been shown to be effective are lung-protective ventilatory strategies and supportive treatment including prophylactic or therapeutic application of antibiotics and restriction of fluid accumulation (Yanhui et al., 2023). There are no pharmaceutical therapies aimed at the underlying pathology have been shown to be effective (Confalonieri et al., 2017).

Neutrophils constitute the major components of the innate immunity. They are the first to respond against a large range of pathogens, and are then recruited to the inflamed tissue, acting by phagocytosis, release of reactive oxygen species (ROS), degranulation (Segal, 2005) and decondensed chromatin fibers coated with antimicrobial proteins, such as histones and neutrophil elastase, which form neutrophil extracellular traps (NETs) to trap and kill bacteria (Brinkmann et al., 2004). However, NETs may be double-edged swords (Kaplan and Radic, 2012), with excessive formation playing an important role in the pathogenesis of ARDS (Porto and Stein, 2016): NETs expand into the pulmonary alveoli, causing lung injury, and induce epithelial and endothelial cell death (Yago et al., 2018). NETs also provoke the formation of immuno-thrombosis (Yago et al., 2018), which is associated with worse ARDS outcomes. There is therefore a sound rationale for strategies that target histones and decrease NET formation in the treatment of ARDS.

Recent studies showed that extracellular histones significantly increased the development of ARDS. In an observational study that included 96 patients with ARDS and 30 healthy volunteers, extracellular histone levels from plasma and bronchoalveolar fluid (BALF) were highly correlated to the severity of ARDS and were significantly higher in non-survivors (Lv et al., 2017). In 52 patients with non-thoracic trauma, high circulating histone levels were associated with an increased incidence of acute lung injury (ALI) and higher Sequential Organ Failure Assessment (SOFA) scores (Abrams et al., 2013). Interestingly, anti-histone antibody therapy protected mice from histone-induced lethality (Lv et al., 2017). Targeting extracellular histones may thus represent a promising therapeutic option for ARDS (Karki et al., 2020).

A polyanion molecule of *β*-O-methyl cellobiose sulfate sodium salt (mCBS.Na), STC3141, has been identified as able to neutralize histones and decrease histone-induced red blood cell (RBC) and platelet aggregation *in vitro* (Meara et al., 2020). Its use was associated with a survival benefit in a rat cecal ligation and puncture model (Meara et al., 2020). This molecule has also been shown to decrease infarct size in a rat cardiac ischemia/reperfusion model (Shah et al., 2021). Beneficial effects in ARDS are therefore anticipated.

Our hypothesis was that STC3141 administration would improve outcomes in a rat model of ARDS. Dexamethasone administration has been shown to increase ventilator-free days and decrease 60-day mortality in patients with ARDS (Villar et al., 2020), so we decided to use dexamethasone as a control for treatment in this study. The reason we chosen this rat model is that it closely reproduces the acute phase of human ARDS (Chimenti et al., 2020).

## 2 Materials and methods

### 2.1 ALI rat model and experimental protocol

All procedures in this study were conducted in accordance with the guidelines of the National Institute of Health and approved by the Animal Protection and Utilization Committee of the JOINN laboratory.

Six-to eight-week-old male Sprague-Dawley (SD) rats (195g–270 g) (Vital River Laboratory Animal Technology Co., Ltd. Zhejiang) were raised in a pathogen-free environment at temperature 20°C–26 °C under a natural light and dark cycle with free access to standard chow and water. A double-hit LPS strategy (LPS strain: O55:B5, Sigma-Aldrich, St. Louis, MO, United States) was used to create the model.

A total of 68 adult male SD rats were randomized to a sham group (Sham, N = 8), in which animals received intraperitoneal injection and intra-tracheal nebulized saline only, or a double-hit LPS group (N = 60). The double-hit LPS consisted of 0.8 mg/kg intraperitoneal injection followed after 16 h by 5 mg/kg intra-tracheal nebulized LPS. For the nebulized LPS or saline, animals were anesthetized by inhalation of isoflurane, then fixed on a 45 inclined rat holder plate. An anesthetic laryngoscope was used to pin the animal’s tongue and expose the glottis. A micro liquid atomizer loaded (HRH-MAG4, produced by Beijing Huironghe Technology Co., Ltd.) with LPS solution or saline was inserted into the trachea, achieving precise and quantified aerosol administration to the lungs. The rats were then placed vertically and rotated for 0.5–1 min to insure a uniform distribution of solution in the lung.

**FIGURE 1:**
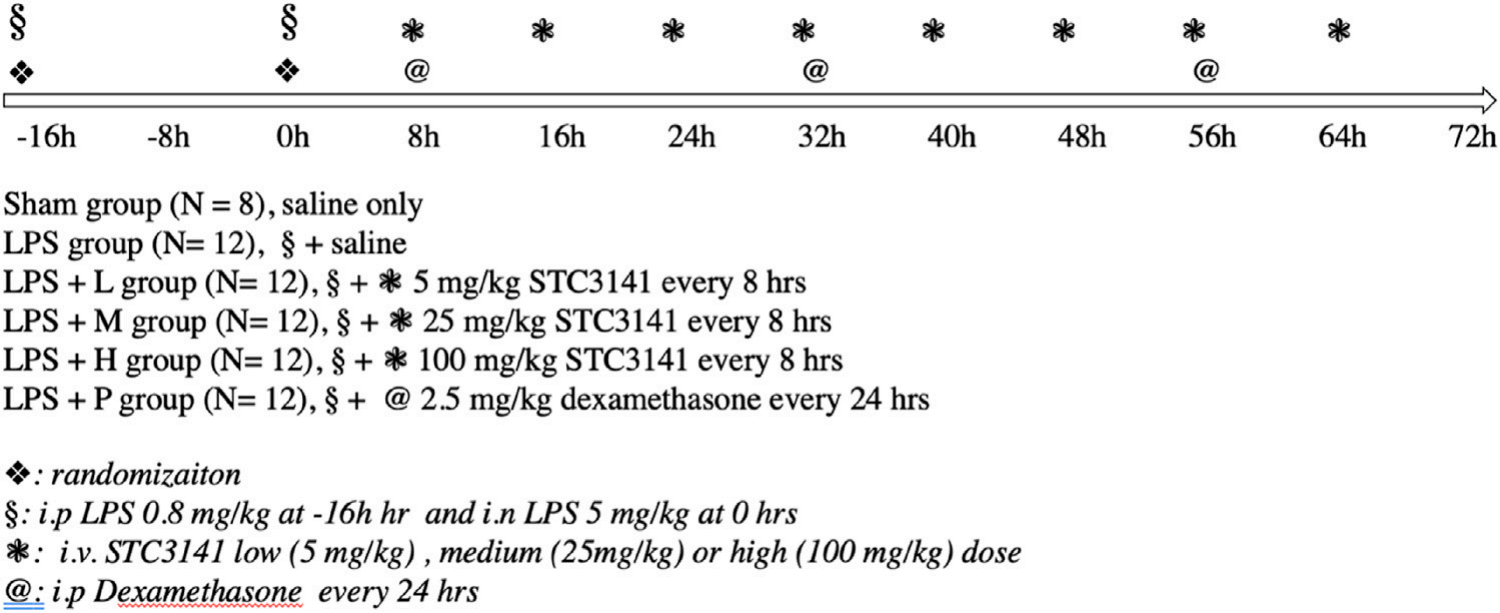
Experimental protocol.

The LPS animals were then randomized to an ARDS control group (LPS, *n* = 12), in which animals received an intravenous injection of saline every 8 h for 64 h; a LPS + L group (*n* = 12), animals received an intravenous injection of 5 mg/kg STC3141 (Grand Medical Pty Ltd.) every 8 h for 64 h; LPS + M group (*n* = 12), animals received an intravenous injection of 25 mg/kg STC3141 every 8 h for 64 h; LPS + H group (*n* = 12), animals received an intravenous injection of 100 mg/kg STC3141 every 8 h for 64 h; or a positive control group (ARDS + D, *n* = 12) in which animals received an intra-peritoneal injection of 2.5 mg/kg dexamethasone (KingYork, Tianjin, China) every 24 h for a total of 56 h (Figure1).

### 2.2 Euthanasia, autopsy, blood sampling, and BALF collection

In accordance with the American Veterinary Medical Association Guidelines (2013), at 72 h after intra-tracheal LPS nebulization, rats were anesthetized with chloral hydrate (350 mL/kg, 100 mg/mL), and then euthanized after collection of 1 mL arterial blood from the abdominal aorta, which was exposed by opening the abdomen along a medioventral line. After the blood sample is collected, the wet ice is temporarily stored. Within 2 h, it is centrifuged at 4°C at 3,000 rpm. The separated plasma is respectively loaded into the cryopreservation tubes, and frozen at—70°C.

To collect BALF, a tracheal cannula was slowly inserted into the trachea through an incision in the left bronchus and fixed in the centripetal direction with threads. A total volume of 3 mL PBS buffer was slowly injected to fill the lung and thereafter gently withdrawn. This procedure was repeated 3 times, for 10 s each time, and the BALF was collected into a centrifuge tube. It was then centrifuged at 4°C with 1,200 g for 10 min. The supernatant was separated and stored at −80°C. The precipitation was resuspended with 1 mL PBS buffer for cell counting. Cell counting and classification of leukocytes in the BALF was performed using an automatic blood cell analyzer.

The middle lobe of the right lung was isolated and weighed to calculate the wet-to-dry ratio. The remaining right lung tissue and bronchi were fixed in 10% neutral buffered formalin solution, paraffin-embedded, sectioned, and hematoxylin-eosin (HE) stained for pathomorphological examination of the lung tissue.

### 2.3 Blood gas and histone measurements in plasma and BALF

At autopsy, a blood gas analyzer (i-STAT ^®^1 Handheld Blood Analyzer (300-G. Abbott Point of Care Inc. United States)) was used to measure PO2 (mmHg), PCO2 (mmHg), pH, and SO2%. Blood samples were collected and centrifuged at 4°C for about 3,000 rpm. The plasma was collected and stored at −70°C. The blood gases was measured while isolated from air. Specifically, during blood gas analysis, a specialized arterial blood sampling needle was used. After blood collection, the needle was inserted into a rubber stopper to isolate it from air. Strict adherence to the operational steps is followed during both blood collection and addition of blood to the blood gas card. The blood gas card was then inserted into the analyzer for testing. The process of blood gas analysis was conducted in a manner that isolated it from air.

Roche’s Cell Death Detection ELISA kit (Cat. No. 11774425001) was used to detect histones. This kit uses Sandwich ELISA to determine histone levels in plasma samples and alveolar lavage fluid, suitable for multiple species such as humans, rats, and mice. I have consulted Roche’s technical support in the early stage, and the histone data results can be directly expressed using the difference between A405 nm and A490 nm. During actual testing, strictly follow the steps required in the manual to perform histone concentration testing.

### 2.4 Histology and inflammation

Lung tissues were fixed, embedded, sectioned, and stained with hematoxylin and eosin (H–E) for histopathology analysis. The lung injury scores were estimated in a blinded manner based on the pathological ALI scoring system adapted from Matute-Bello study (Matute-Bello et al., 2011). Briefly, several parameters—such as A: presence of neutrophils in the alveolar space; B: neutrophils in the intraseptal space; C: hyaline membrane; and D: protein debris—were evaluated in the histological sections. Score = [(20 x A) + (14 x B) + (7 x C) + (7 x D)]/(number of fields x 100).

The levels of myeloperoxidase (MPO) and Histone H3 (H3) in plasma were detected by using an enzyme linked immunosorbent assay (ELISA) using commercially available kits according to the manufacturer’s instructions (USCN life Science Inc., Wuhan, China).

### 2.5 Lung wet-to-dry weight

The right middle lobe was weighed, then heated to 60°C for 72 h and weighed again. The wet-to-dry weight ratio was calculated using the formula: lung wet-to-dry ratio = right middle lobe wet weight/dry weight.

### 2.6 Data collection and statistical analysis

All experimental data were statistically analyzed by using GraphPad Prism (version 8.0, United States) and were expressed as the mean ± standard error of mean (SEM). We employed an unpaired Student’s t-test to assess the statistical significance of differences between two groups. For comparison of more than two groups, multiple comparisons by one-way analysis of variance (ANOVA) followed by post-hoc Tukey’s multiple comparison tests. Level of statistical significance was set at *p* < 0.05.

## 3 Results

### 3.1 STC3141 attenuated circulating histone levels and pulmonary edema

**FIGURE 2:**
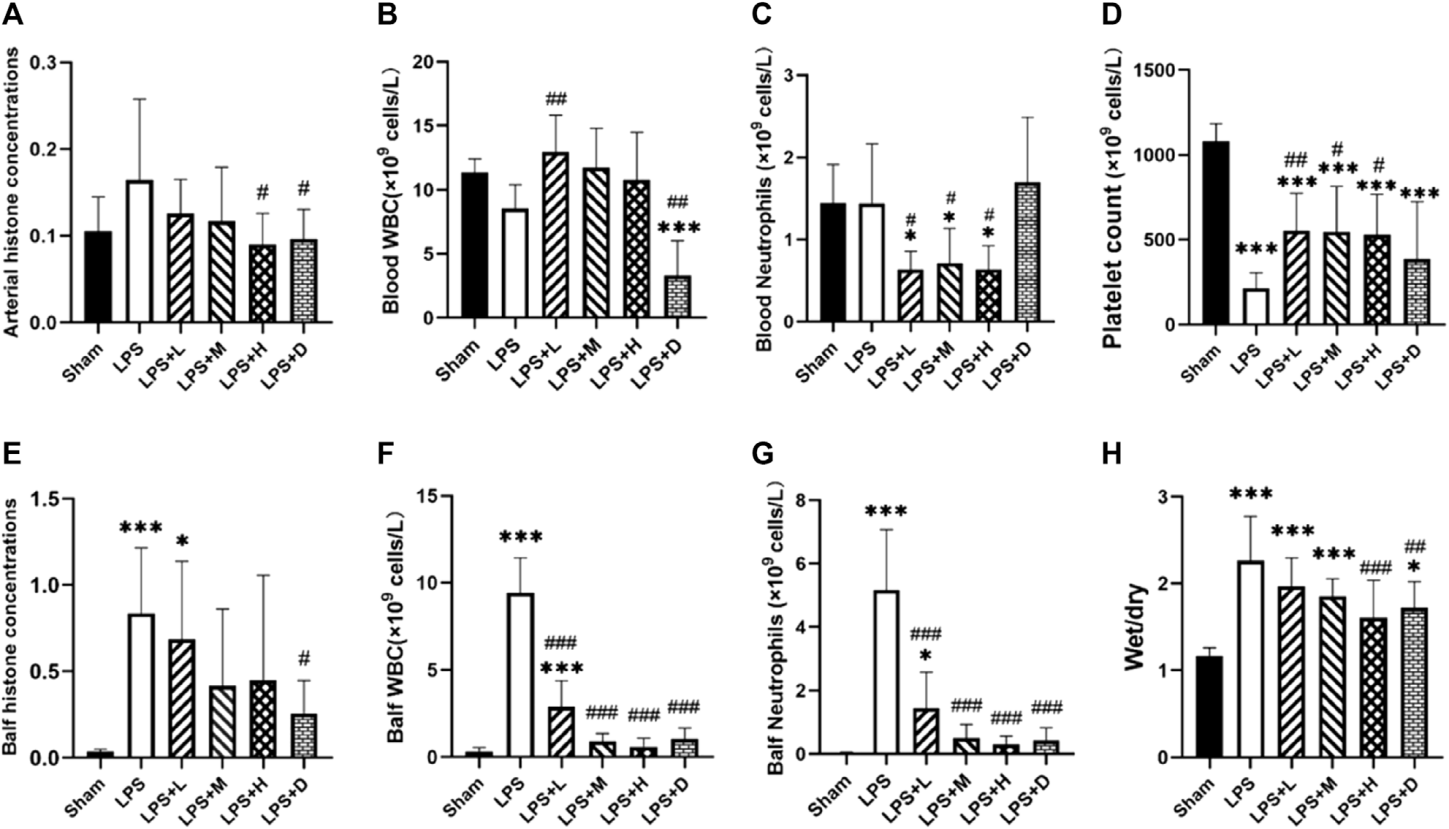
Arterial and bronchoalveolar lavage fluid (BALF) **(A, E)** histone concentrations **(B, F)** white blood cell (WBC) **(C, G)** neutrophil **(D)** platelet counts, and **(H)** lung wet-to-dry ratio in the different groups: data expressed as mean ± standard deviation; *p < 0.05, ***p < 0.001 *versus* the sham group; #*p* < 0.05, ##*p* < 0.01, and ###*p* < 0.001 *versus* the LPS group.

All the 68 animals survived to 72 h after the LPS second hit (Figure 1). The levels of BALF histone concentrations were significantly higher in the LPS group as compared with the sham group, but there was no difference in circulating histone levels between the two group (Figure 2A, E). Meanwhile, both the LPS + H and LPS + D groups had significantly lower circulating histone levels than the LPS only group, and the LPS + D group also had lower BALF histone concentrations than the LPS only group (Figures 2A, E).

The ratio of wet/dry weight of lung tissue was significantly higher in the all LPS groups as compared with the sham group (Figure 2H). However, both the LPS + H and LPS + D groups had a significantly lower lung wet-to-dry ratio than that of the LPS only group (Figure 2H).

### 3.2 STC3141 reduced lymphocyte recruitment and neutrophil infiltration

The graph shows that compared with the LPS only group, the LPS + D group had significantly lower white blood cell (WBC) counts in the blood and BALF; The LPS + L, +M, and +H groups significantly reduced the count of WBC in the BALF and did not reduce it in the blood (Figure 2B–F). Compared with the LPS only group, the LPS + L, +M, and +H groups significantly reduced the count of neutrophils in the blood and BALF; The LPS + D group significantly reduced the count of neutrophils in the BALF, but have no influence in the blood (Figure 2C–G). Meanwhile, compared with sham group, the LPS only group increased the counts of WBC and neutrophils in the BALF, but have no influence in the blood.

Furthermore, the total number of platelet counts were significantly lower in the all LPS groups as compared with that of the sham group (Figure 2D); Compared with the LPS only group, the counts of platelet were significantly higher in the LPS + L, +M and +H groups; There was no significantly difference in platelet counts between the LPS only group and LPS + D group (Figure 2D).

### 3.3 STC3141 decreases lung injury score and inflammatory cytokine production

**FIGURE 3:**
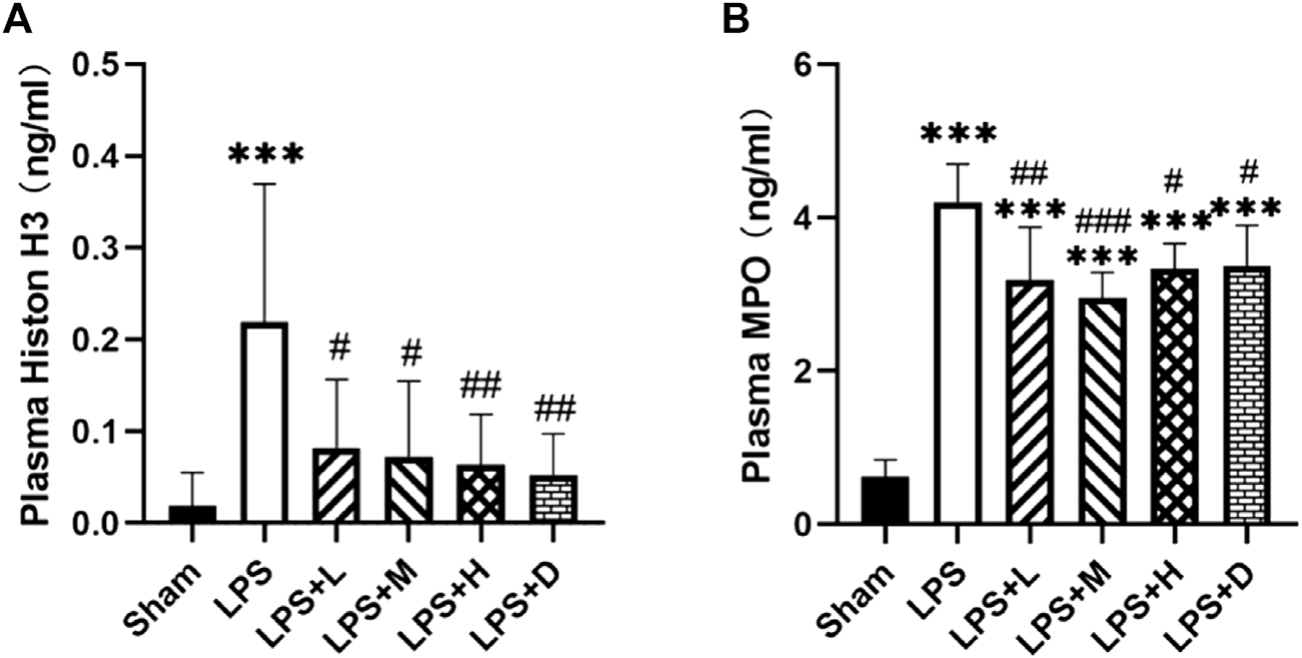
STC3141 reduces ALI-induced inflammatory cytokines in plasma. **(A)** Histone H3, and **(B)** MPO. Data are presented as means ± SEM, *n* = 7–8. ***p < 0.001 *versus* the sham group; #*p* < 0.05, ##*p* < 0.01, and ###*p* < 0.001 *versus* the LPS group.

To further confirm the effect of neutrophil extracellular traps (NETs), we analyzed the expression of MPO, a classical marker of neutrophil activation, and the expression of Histone H3 (H3), a classical marker of histone activation. The graph shows that the expression of MPO and H3 were significantly higher in the LPS only group as compared with that of the sham group; However, the total number of MPO and H3 were significantly lower in the LPS + L, +M, +H and +D groups than in the LPS only group (Figure 3A, B).

**FIGURE 4:**
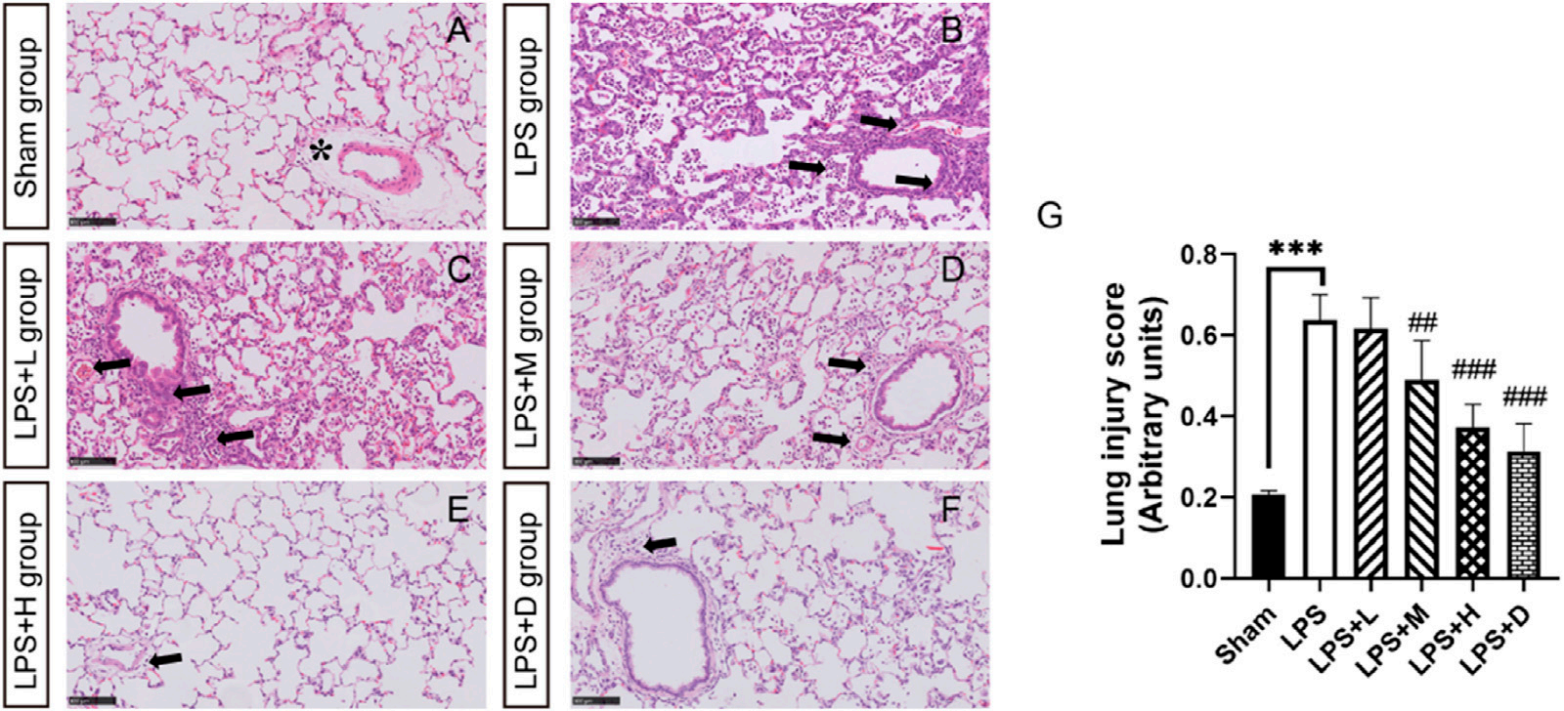
STC3141 improves lung injury scores for LPS-induced ALI in rats. Representative histology of the right upper lobe in the different groups **(A)** in sham group **(B)** LPS group **(C)** LPS + L group **(D)** LPS + M group **(E)** LPS + H group **(F)** LPS + D group. **(G)** Lung injury score. All images are taken after HE staining and amplified 100X. Data are presented as means ± SEM, *n* = 7. ***p < 0.001 *versus* the sham group; ##*p* < 0.01, and ###*p* < 0.001 *versus* the LPS group. Note that the artifactual expansion of the peribronchial tissue (asterisk), which commonly results from tissue processing, does not represent edema. Lung inflammation, with neutrophil-based inflammatory cells (alveolar cavity, blood vessel, alveolar wall), alveolar wall thickening, bleeding in the alveoli (arrows).

Histopathological examination showed lung inflammation, with neutrophil-based inflammatory cells (alveolar cavity, blood vessel, alveolar wall), alveolar wall thickening, bleeding in the alveoli (with or without hemoglobin crystals). The degree of inflammation was significantly less in the LPS + M, +H and +D groups than in the LPS only group (Figure 4G). Inflammation in the LPS + L, +M and +H animals showed a dose-dependent response: as the dose increased, the degree of severity decreased. Representative histological findings in the right upper lobe in the different groups are shown in Figure 4A–F).

### 3.4 STC3141 mitigated LPS-induced acute lung injury

**FIGURE 5:**
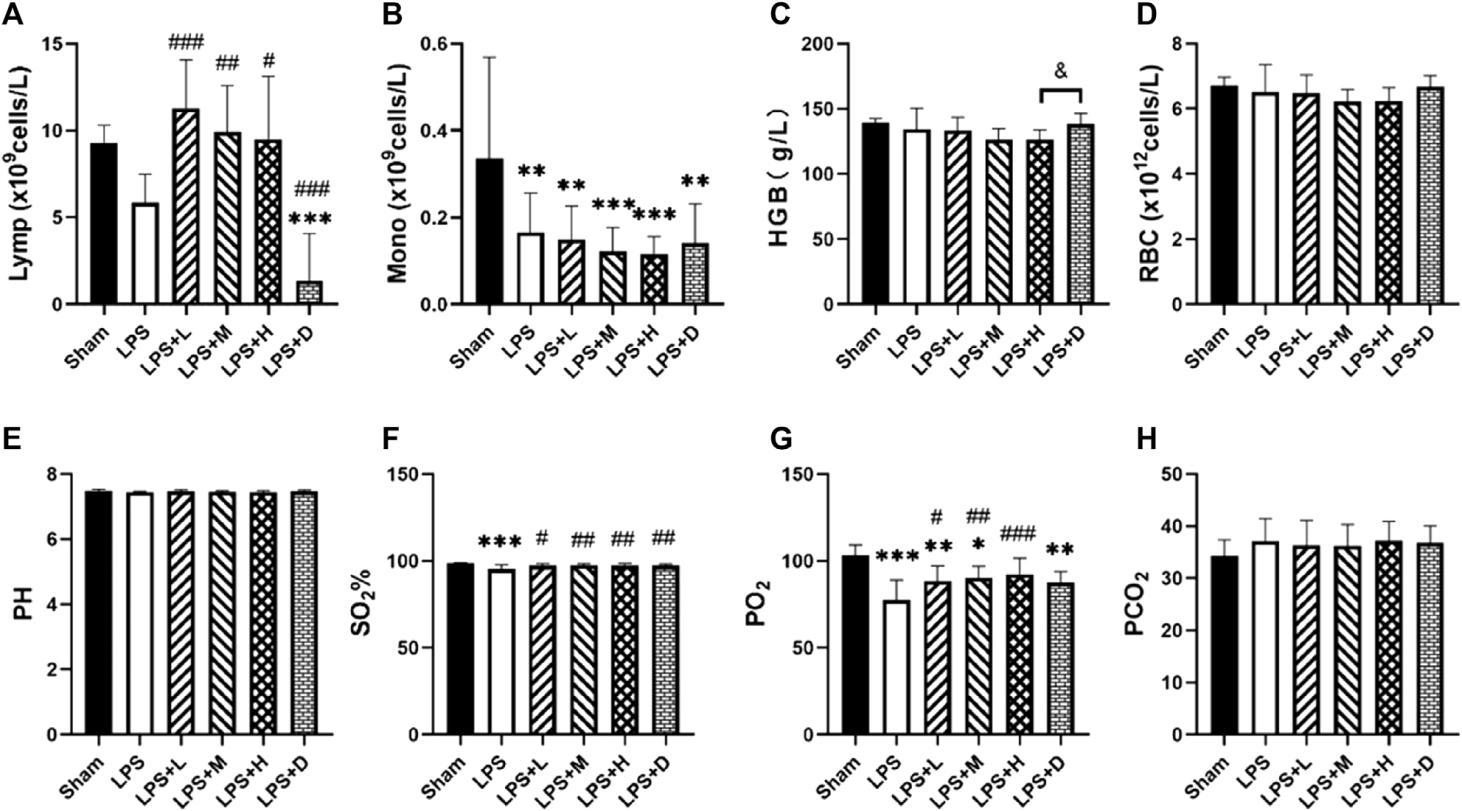
**(A)** Lymphocyte (Lymp) **(B)** monocyte (Mono) counts **(C)** hemoglobin (Hb) concentration and **(D)** red blood cell (RBC) in the blood **(E)** arterial pH **(F)** SO2% **(G)** PO2, and **(H)** PCO2in the different groups. *p < 0.05, **p < 0.01, and ***p < 0.001 *versus* the sham group; #*p* < 0.05, ##*p* < 0.01, and ###*p* < 0.001 *versus* the LPS group; &*p* < 0.05 *versus* the LPS + H group.

The animals in the LPS group developed acute lung injury, with arterial blood PO2 (77.5 ± 11.4 mmHg *versus* 103.3 ± 5.9 mmHg) and oxygen saturation (95.3% ± 2.5% *versus* 98.4% ± 0.5%) significantly lower in the LPS only group than in the sham group (Figure 5). Comparison between LPS groups, the arterial PO2 was significantly higher in the LPS + L, +M, and +H groups than in the LPS only group; the oxygen saturation was significantly higher in the LPS + D group than in the LPS only group. There were no statistical differences in PCO_2_, RBC or pH among groups (Figure 5).

## 4 Discussion

The main findings of the current study are: 1) that a double-hit of LPS induced a model of ALI in rats as shown by the presence of inflammation, decreased oxygenation, lung edema, and histological changes, representing clinical ARDS findings; 2) neutralization of extracellular histone with low, medium and high dose STC3141 significantly decreasing circulating and BALF WBC and neutrophil counts, improving lung oxygenation and decreasing lung inflammation; 3) high dose STC3141 seems to have a similarly beneficial effect on oxygenation and lung edema formation to dexamethasone administration in this ALI model.

The pathological study of ARDS showed that there were a large number of proteins and inflammatory cells in edema fluid accumulated in alveoli and interstitial lung, among which neutrophils (Grommes and Soehnlein, 2011) were dominant. Activation and recruitment of neutrophils are considered to play a key role in the development of ARDS. Neutrophils are the first cells to be recruited to inflammatory sites. They have a strong antibacterial mechanism included oxidizing agent, protease and cationic peptide. However, under pathological conditions, uncontrolled release of these antibacterial substances outside the cell will destroy the host tissue (Grommes and Soehnlein, 2011). With the deepening of research on neutrophils, more and more researchers have found that neutrophil extracellular traps (NETs) are closely related to the inflammatory injury of ARDS. Excessive neutrophils played a pathogenic role in acute lung injury of influenza pneumonia, and NETs formed by neutrophils could cause alveolar-capillary injury (Narasaraju et al., 2011). Since then, animal experiments and clinical trials have reported that NETs can cause lung tissue damage and promote ARDS. NETs (Lefrançais et al., 2018; Li et al., 2018; Mikacenic et al., 2018) can be detected in alveoli and plasma of ARDS patients. The level of NETs is related to the poor prognosis of patients (Zhu et al., 2018). These results indicate that NETs is involved in the inflammatory reaction in the pathogenesis of ARDS.

Since NETs was first proposed by Brinkmann et al., in 2004 (Brinkmann et al., 2004), the related research has made new progress continuously. At first, NETs was only regarded as a complex function of innate immune system to fight against invading microorganisms. Later, people clearly realized that NETs had a far-reaching role. NETs is involved in a series of pathophysiological mechanisms from inflammation to thrombosis, When the generation or elimination of NETs is not fully controlled, they will have a serious impact on the organism. NETs is composed of filamentous DNA and a large number of protein particles attached to it, among which histone is dominant, accounting for about 70% of NETs-related proteins (Urban et al., 2009), besides neutrophil elastase and myeloperoxidase. When DNA as the backbone of NETs is degraded by DNAse, Histones separated from DNA will exist in free form. More and more studies have shown that histone, which plays a bactericidal role in NETs, is an important factor (Jamison and Grailer, 2014) leading to lung injury in ARDS. Free histones and extracellular histones contained in NETs are highly cytotoxic to alveolar epithelial cells and endothelial cells, which can directly lead to the death of these cells (Saffarzadeh et al., 2012). It can continue to induce neutrophils to produce NETs (Nakazawa et al., 2017; Shrestha et al., 2019), so that a large number of neutrophils will be recruited into the lungs, thus forming the amplification effect of inflammatory waterfall, further aggravating the degree and expanding the scope of lung injury. After these necrotic cells disintegrate, histones in the nucleus will also disperse outside the cells, damaging surrounding cells and tissues, and further expanding the damage (Jamison and Grailer, 2014; Nakazawa et al., 2017). Therefore, studying NETs, especially extracellular histone, as a target may provide an effective method for ARDS treatment.

At present, many studies have tried to treat ARDS by targeting NETs. DNA enzyme can destroy the three-dimensional structure of NETs by degrading DNA in NETs, and it has shown good efficacy in some ARDS animal models (Caudrillier et al., 2012). Activated protease C (APC), which was once listed as a serum protease to degrade extracellular histone, also showed good results in animal experiments. However, in human body, because APC has anticoagulant effect at the same time, when its concentration has not reached the ideal concentration for removing extracellular histone, However, it has shown obvious anticoagulant effect, which leads to the increase of bleeding risk, and it is difficult to balance the efficacy and safety risk, which eventually leads to the withdrawal of APC from the market. In addition, some studies have shown that negatively charged drugs, including heparin and albumin, can combine with extracellular histone through electric charge, thus reducing the toxic effect of histone (Lam et al., 2013; Wildhagen et al., 2014). Treat with histone antibody, It can also effectively neutralize the toxicity of extracellular histone (Xu et al., 2009; Wildhagen et al., 2014; Kusano et al., 2015).

LPS exposure may lead to lung inflammation. An LPS stimulated rat model is a standard experimental ALI and ARDS model and widely used for new drug development for this condition (Martin and Silverman, 1992; Uhlig and Kuebler, 2018; Liang et al., 2020). ALI and ARDS could be induced in rats by intratracheal, intraperitoneal administration of LPS or by an LPS double-hit method. Intraperitoneal injection with LPS can induce indirect ALI while intra-tracheal nebulized LPS is used to induce direct ALI. Recent studies have reported that double-hit model is more stable and reliable, closer to the diagnostic criteria of ARDS in humans and better for mimicking the pathological process of ARDS (Chen et al., 2010). Moreover, intra-tracheal nebulized LPS causes robust neutrophilic alveolitis and is used to evaluate both the early and resolution phases of ALI (Yanhui et al., 2023). In our study, LPS animals exhibited decreased oxygenation, lung inflammation (intra-alveolar leukocytes, histological injury), and higher histone concentrations than sham animals. Meanwhile, targeting extracellular histones using STC3141 significantly decreased WBC and neutrophil counts not only in the circulation, but also in the BALF, which suggests it may interrupt NET formation, thereby ameliorating the impaired microcirculation and improving lung function. However, low and medium dose STC3141 treatment did not decrease circulating histone levels, but high dose STC3141 and dexamethasone did. This dose-dependent effect is reflected by the findings of similar lung dry/wet ratio in the high dose and dexamethasone groups: improved oxygenation and decreased lung inflammation and edema formation. STC3141 administration significantly decreased histone-induced RBC and platelet aggregation *in vitro*, and histone-induced tissue injury, thrombocytopenia and anemia in mice (Meara et al., 2020). These preclinical findings could help to explain the observations in ARDS patients, with a significant increase in plasma histones in mild, moderate, and severe ARDS and significantly higher plasma histones in non-survivors than in survivors (Lv et al., 2017). Furthermore, in ARDS patients with a good prognosis, plasma histone levels decreased after admission whereas they increased in those with a poor prognosis (Lv et al., 2017). All these findings support the concept of histone neutralization therapy in ARDS.

The study has several limitations. First, LPS is well known to induce inflammation rather than infection, and the model therefore does not fully mimic the disease kinetics in ARDS (Xu et al., 2009). Second, the young healthy animals used in the current study are different from the typical ARDS patient with comorbidities. Third, the shortage of direct evidence immunohistochemistry results of NETs from lung with STC3141 due to limited label technology.

## 5 Conclusion

In the current LPS double-hit rat model of ALI, high dose STC3141 showed similar effects to dexamethasone therapy, including significantly decreased circulating histone concentrations, improved oxygenation, and decreased lung edema formation. Clinical trials are necessary to identify the effects of histone neutralization in ARDS patients.

## Data Availability

The raw data supporting the conclusion of this article will be made available by the authors, without undue reservation.
